# Review of Recent Attempts to Judge Pancreatic Activity by Clinical Tests

**Published:** 1912-10

**Authors:** 


### ABSTRACTS

---

Review of Recent Attempts to Judge Pancreatic Activity by Clinical Tests.—By Dr. Leech (*The Practitioner,* November, 1911).

The glutoid and salol tests are of little actual value; the nuclear test is doubtful. The presence of voluminous light-colored stools, undigested meat fibers, and of fat in large quantity, especially of neutral fat, are valuable evidence of pancreatic disease, but of advanced pancreatic disease either actively destructive or blocking the duct and not of fine changes which frequently cause dyspepsia. The value is enhanced if a Schmidt's diet is given. The unfavorable verdict in numerous recent articles on Cammidge's test is disappointing; the test cannot be said to have fulfilled the hope built up on it. The future of various ferment tests seem good, especially those for amylase and trypsin in the feces, and perhaps of amylase in the urine. The worth of the tests is all the greater where the value of ferment in urine and feces can be compared. A large field of work lies open on this subject but the present clinical value is small. Gross changes alone are indicated; we have yet no sure test for fine pancreatic changes.

